# Osteoarthritic chondrocytes undergo a glycolysis-related metabolic switch upon exposure to IL-1b or TNF

**DOI:** 10.1186/s12964-023-01150-z

**Published:** 2023-06-14

**Authors:** Anais Defois, Nina Bon, Alexandre Charpentier, Melina Georget, Nicolas Gaigeard, Frederic Blanchard, Antoine Hamel, Denis Waast, Jean Armengaud, Ophelie Renoult, Claire Pecqueur, Yves Maugars, Marie-Astrid Boutet, Jerome Guicheux, Claire Vinatier

**Affiliations:** 1 Nantes Université, Oniris, CHU Nantes, INSERM, Regenerative Medicine and Skeleton, RMeS, UMR 1229, 44000 Nantes, France; 2 Département Médicaments Et Technologies Pour La Santé (DMTS), Université Paris-Saclay, CEA, INRAE, SPI, Bagnols-Sur-Cèze, 30200 France; 3 Nantes Université, INSERM, CNRS, CRCI2NA, F-44000 Nantes, France; 4 Centre for Experimental Medicine & Rheumatology, William Harvey Research Institute and Barts and The London School of Medicine and Dentistry, Queen Mary University of London, London, UK

**Keywords:** Metabolism, Inflammation, Osteoarthritis, Chondrocytes

## Abstract

**Background:**

Osteoarthritis is an age-related disease that currently faces a lack of symptomatic treatment. Inflammation, which is mainly sustained by pro-inflammatory cytokines such as IL-1b, TNF, and IL-6, plays an important role in osteoarthritis progression. In this context, pro-inflammatory cytokines are widely used to mimic the inflammatory component of osteoarthritis in vitro. However, the therapeutic failures of clinical trials evaluating anti-cytokines drugs highlight the lack of overall understanding of the effects of these cytokines on chondrocytes.

**Methods:**

Here, we generated a comprehensive transcriptomic and proteomic dataset of osteoarthritic chondrocytes treated with these cytokines to describe their pro-inflammatory signature and compare it to the transcriptome of non-osteoarthritic chondrocytes. Then, the dysregulations highlighted at the molecular level were functionally confirmed by real-time cellular metabolic assays.

**Results:**

We identified dysregulation of metabolic-related genes in osteoarthritic chondrocytes but not in non-osteoarthritic chondrocytes. A metabolic shift, toward increased glycolysis at the expense of mitochondrial respiration, was specifically confirmed in osteoarthritic chondrocytes treated with IL-1b or TNF.

**Conclusion:**

These data show a strong and specific association between inflammation and metabolism in osteoarthritic chondrocytes, which was not found in non-osteoarthritic chondrocytes. This indicates that the link between inflammation and metabolic dysregulation may be exacerbated during chondrocyte damage in osteoarthritis.

Video Abstract

**Supplementary Information:**

The online version contains supplementary material available at 10.1186/s12964-023-01150-z.

## Introduction

Osteoarthritis (OA), the most common joint disease in humans, affects over 500 million people worldwide, including a significant proportion of the elderly population, resulting in pain and disability [1]. The incidence of OA and its socioeconomic costs, representing between 1% and 2.5% of the gross domestic product in developed countries [2], are expected to rise with the increase in global life expectancy. However, there is currently no cure for OA, and management of this debilitating condition only consists of diet and lifestyle changes, pain management, and prosthetic joint replacement at late stages. Thus, the development of disease-modifying osteoarthritis drugs (DMOADs) is a major challenge and requires a better understanding of OA pathophysiology.

Although long considered to be only a cartilage disease, it is now widely recognized that OA affects all joint tissues through inflammatory and degenerative processes [3]. Chronic low-grade inflammation appears to play a key role in OA development, and joint inflammation is largely sustained by the synovium. Indeed, synovitis correlates with the severity of OA [4], and synovium changes can arise before the visible onset of cartilage degradation, thus highlighting the significance of synovial inflammation in OA. There is nonetheless a close relationship between the synovium and cartilage during OA. Chondrocytes, through the production of damage-associated molecular patterns, undergo and support local inflammation of the synovial tissue by producing and releasing pro-inflammatory cytokines. In this chronic pro-inflammatory environment, chondrocytes acquire a pro-catabolic and pro-inflammatory phenotype, leading to the production of a plethora of proteins, such as proteases and pro-inflammatory cytokines [5, 6].

Moreover, IL-1β (IL-1b), TNF, and, to a lesser extent IL-6, the three main pro-inflammatory cytokines found in abundance in OA synovial fluid [7], are commonly used to mimic pro-catabolic and pro-inflammatory chondrocyte phenotypes in vitro. Thus, several mechanisms are altered by these cytokines. They decrease the capacity of chondrocytes to produce extracellular matrix, while broadly enhancing their catabolic activity [8], ultimately leading to extracellular matrix (ECM) degradation. Moreover, pro-inflammatory stimulation of chondrocytes results in IL-1b, TNF, and IL-6 overexpression [5], ultimately promoting deleterious mechanisms involved in OA, such as cellular senescence [9] and oxidative stress [10].

Given the crucial role played by these cytokines in perpetuating the low-grade inflammation characteristic of OA, it appeared relevant to evaluate the effect of anti-TNF (e.g., etanercept, adalimumab), anti-IL-1 (e.g., anakinra, lutikizumab), and anti-IL-6 (tocilizumab) drugs as DMOADs. According to the regulatory requirements, a DMOAD must improve symptoms, such as pain and/or disability, and stop or at least slow the loss of joint space width [11]. However, despite promising results in OA animal models [12–14] and efficacy in other types of inflammatory rheumatisms, all of these anti-cytokine drugs failed to relieve pain in patients with OA [15]. Specifically, the recently developed dual anti-IL1α/β molecule lutikizumab was tested in OA patients with synovitis and demonstrated only limited improvement in pain scores and synovial inflammation [16]. Similarly, etanercept [17] and tocilizumab [15] failed to relieve pain in patients with hand OA. Thus, given the therapeutic failure of individual targeting of IL-1b, TNF, or IL-6 signaling, a thorough investigation of shared dysregulated pathways may allow the identification of common clinically relevant therapeutic targets for the development of higher-impact therapies for OA.

Several metabolic dysregulations have been described in an OA context. Among them, mitochondrial defects and alterations in glycolytic enzymes are described in OA chondrocytes [18–20]. Interestingly, promising new therapeutic compounds currently under clinical evaluation such as sprifermin (recombinant human FGF18) have recently shown a protective effect against OA-related metabolic alterations in vitro, evidenced by an improvement of mitochondrial function and inhibition of ROS production [21]. It is also increasingly recognized that there is a close link between inflammation and metabolic adaptation [22]. In inflammatory arthritis, deleterious metabolism changes receive increasing attention, particularly in synovial cells [23]. Moreover, impairment of adenosine metabolism by methotrexate, a drug used in rheumatoid arthritis treatment [22] has been shown to significantly improve pain and stiffness in OA patients, while drugs targeting pro-inflammatory cytokines fail to improve it [27], thus confirming that metabolism should be considered a key player in the clinical response to treatment. Nevertheless, the underlying mechanisms of these metabolic dysregulations in the pathophysiology of OA, although present, are still poorly understood and the link between chronic low-grade inflammation and these dysregulations in cartilage remains to be explored.

Here, multi-omics analyses were performed in articular chondrocytes from OA joints (OACs) to decipher common transcriptional and proteomic changes in response to IL-1b, TNF, and IL-6 pro-inflammatory stimuli. To unravel whether the common inflammatory signature identified is specific to OACs, the transcriptome of chondrocytes from non-osteoarthritic joints (NCs) following pro-inflammatory stimuli was used as an internal control and compared to the transcriptome of OACs.

In this study, we highlighted that the metabolic shift usually observed in chondrocytes upon exposure to pro-inflammatory stimuli was a specific feature of OA chondrocytes and was not found in non-OA chondrocytes. This insensitivity of non-OA chondrocytes to metabolic alteration may open new avenues for understanding the dysregulated mechanisms that occur during OA.

## Results

### Transcriptomic analysis revealed overlapping but non-redundant NC and OAC pro-inflammatory responses

**Fig. 1:**
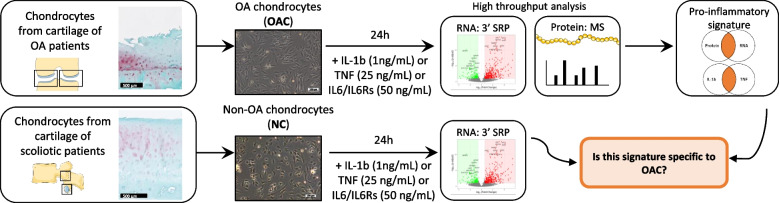
Overview of the study design. OA chondrocytes (OACs) and non-OA chondrocytes (NCs) were treated for 24 h with or without pro-inflammatory cytokines. A multi-omic (RNA 3’ SRP and protein MS) approach was used in OACs to identify a pro-inflammatory signature. Then, NC transcriptomics data were compared to the OACs pro-inflammatory signature to define disease-specific expression features. Scale bar histology: 500 µm, Scale bar cells culture: 100 µm

**Fig. 2:**
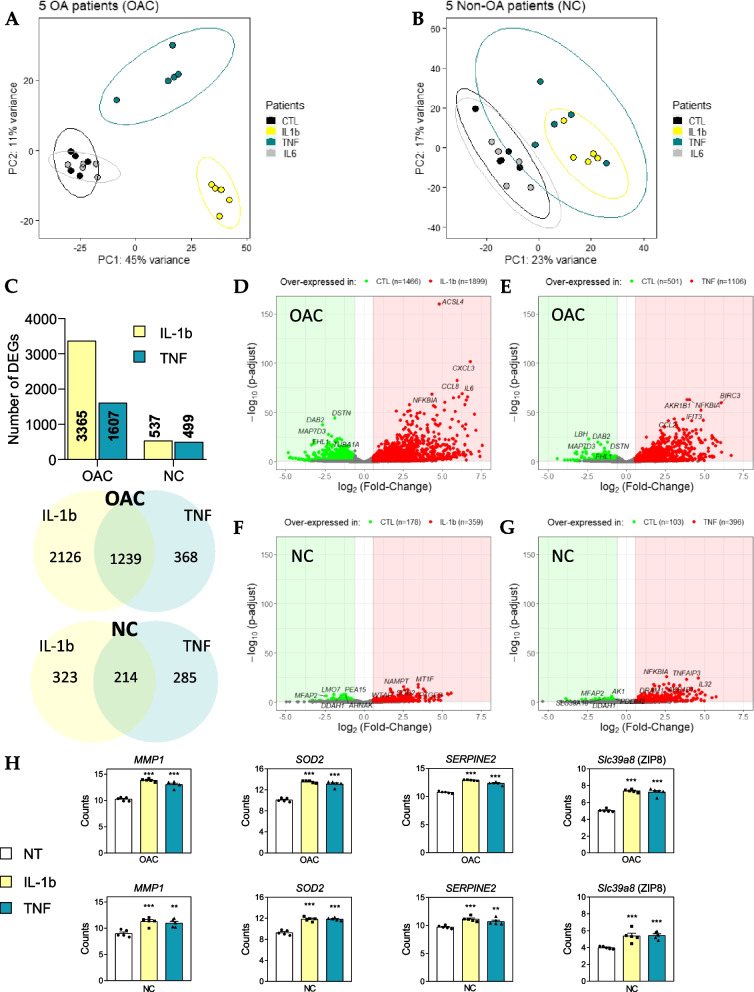
OAC and NC RNA-seq data. (**a**, **b**) Principal component analysis (PCA) was performed on the expression data of OAC (**a**) and NC samples (**b**). The first two eigenvalues were plotted with data ellipses for each treatment. (**c**) Summary of the number of DEGs in each condition and DEGs common between IL-1b and TNF treatments highlighted by Venn diagrams. (**d**, **e**, **f**, **g**) Volcano plots representing DEGs (p-adjust < 0.10; log2(Fold-Change) >|0.58|) in OACs (**d**, **e**) and NCs (f, g) in response to 1 ng/mL IL-1b (d, f) or 25 ng/mL TNF (e, g). (**h**) Histograms representing normalized *MMP1*, *SOD2*, *SERPINE2*, and *Slc39a8* (ZIP8) gene counts in OACs and NCs; **P < 0.01 and ***P < 0.001, versus untreated condition using multiple t-tests with Benjamini & Hochberg correction; *N* = 5 per group. Values are expressed as means ± SEM

Following RNA-sequencing (Fig. 1), OACs principal component analysis (PCA) showed that the samples treated with either IL-1b or TNF significantly clustered away from the untreated samples (Fig. 2A). By contrast, samples treated with IL-6 overlapped with untreated samples. Interestingly the sample segregation observed on NCs PCA was not as distinct as on OACs PCA, exhibiting only significant segregation of IL-1b-treated samples from untreated ones (Fig. 2B). Altogether these data suggest a more pronounced pro-inflammatory response by OACs compared to NCs.

As expected from the PCAs, very few differentially expressed genes (DEGs) were identified between IL-6-treated and untreated samples in OACs (56 DEGs), and no DEGs were identified in NCs (data not shown). We identified 3365 DEGs in response to IL-1b and 1607 DEGs in response to TNF in OACs, while only 537 and 499 DEGs were found in response to IL-1b and TNF, respectively, in NCs (Fig. 2C). Indeed, volcano plots indicated that the overall responses of OACs (Fig. 2D-E) to either IL-1b or TNF were greater compared to NCs (Fig. 2F-G). Moreover, OACs exhibited a higher number of DEG in response to IL-1b (3365 DEGs) than to TNF treatment (1607 DEGs). Nevertheless, 78% of the DEGs identified in response to TNF (1239/1607) were common to those identified in response to IL-1b, highlighting strong redundancy of the TNF response with that of IL-1b (Fig. 2C).

Despite the overall differences observed between NC and OAC responses, 408 DEGs in response to IL-1b and 411 DEGs in response to TNF were found to be common in both cell types (Figure S1A, B). Among these common DEGs overexpressed in both cell types in response to IL-1b and TNF, there were several key players of OA, such as *MMP1* [24]; *SOD2* [25]*, SERPINE2* [26, 27]*,* and *Slc39a8* [28] (Fig. 2H).

Nevertheless, among the 1249 DEGs identified as common to both cytokines in OACs, the DEGs of interest, 1079 (≈86%) were specific to OACs and warranted further examination.

### Multi-omics analysis identified a canonical pro-inflammatory signature in OACs

**Fig. 3:**
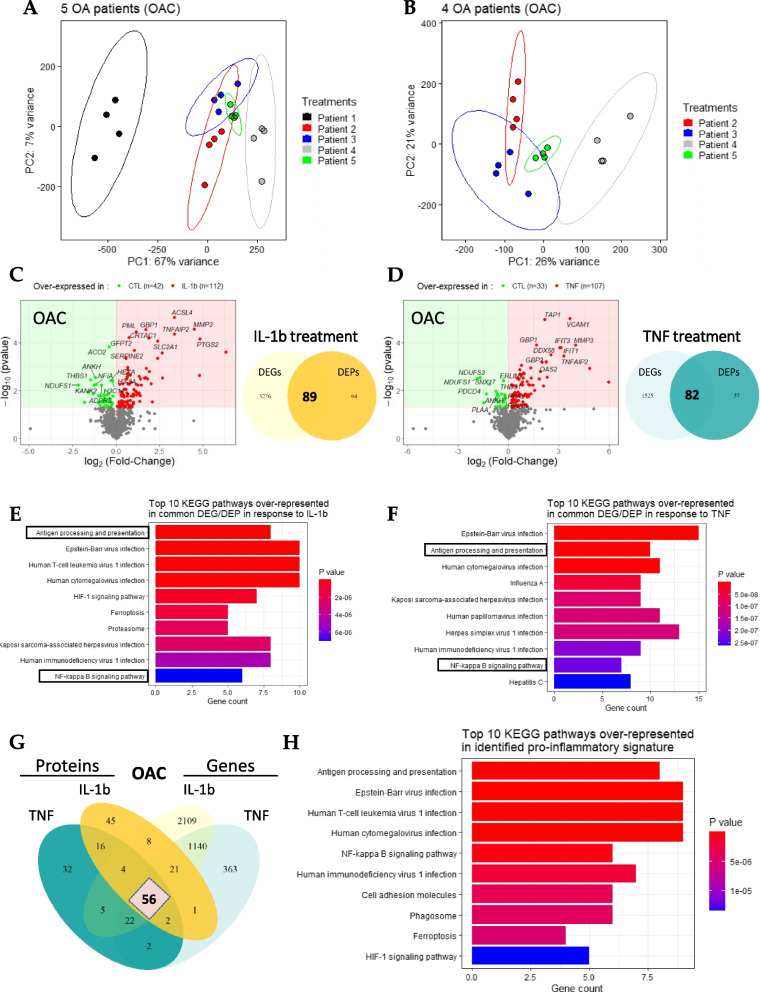
Definition of the canonical pro-inflammatory signature in OACs. (**a**, **b**) PCAs performed on the protein expression data from (**a**) 5 OA patients or (**b**) the 4 selected patients. The first two eigenvalues were plotted with data ellipses for each patient. (**c**, **d**) Volcano plots representing DEPs (*p*-value < 0.05; |log2(Fold-Change)|> 0) and Venn diagram highlighting DEPs and DEGs matched in OAC in response to (**c**) 1 ng/mL IL-1b, or (**d**) 25 ng/mL TNF compared to the untreated condition. (**e**, **f**) KEGG enrichment analysis based on DEGs/DEPs in response to (**e**) IL-1b or (**f**) TNF. (**g**) Venn diagram highlighting 56 DEGs/DEPs common between IL-1b and TNF responses. (**h**) KEGG enrichment analysis using the list of 56 matched DEPs/DEGs

To decipher the specific OAC signature in response to pro-inflammatory stimuli and to complete the description of OACs upon exposure to pro-inflammatory stimuli, in addition to the 3’ SRP analysis, we performed tandem mass spectrometry analysis of the proteins extracted from OAC lysates (5 OA patients) treated the same way as the samples described previously (Fig. 1). This analysis validated and quantified 2688 proteins and, for the entire dataset, we were able to assign 794,053 MS/MS spectra, or an average of 26,468 per sample. The PCA analysis of proteomic samples revealed one significant outlier patient despite the absence of previously obvious differences with other samples (Fig. 3A, in black). Following the removal of this patient from our analysis, the variability between the different samples was drastically decreased, as illustrated by the percentage of variance associated with the first component (PC1), which changed from 67% (*N* = 5) to 26% (*N* = 4) (Fig. 3B). Similar to the transcriptomic results, a low number (52) of differentially expressed proteins (DEPs) were identified in samples treated with IL-6 (data not shown). In light of the small number of DEGs and DEPs identified in response to IL-6, our subsequent analyses focused only on samples treated with either IL-1b or TNF. Interestingly, whereas a significant difference in the number of DEGs in response to IL-1b (3365) or TNF (1607) was observed at the transcriptomic level, at the proteomic level a similar number of DEPs was found in response to IL-1b (154 DEP) or TNF (140 DEPs) (Fig. 3C, D). Among these identified DEPs, 89 (Fig. 3C) and 82 (Fig. 3D) DEPs matched with identified DEGs in response to IL-1b or TNF, respectively. The KEGG pathways analysis based on these common DEGs/DEPs revealed key over-represented pathways such as “Antigen processing and presentation” and “NF-kB signaling pathway” that are shared by both the IL-1b and TNF responses (Fig. 3E, F).

Thereafter, to define the pro-inflammatory signature in OACs, we mainly focused on the similarities between the IL-1b and TNF responses at both the transcriptomic and proteomic levels. With these criteria, we identified 56 matched DEPs/DEGs found in both the IL-1b and TNF responses, which determined a canonical pro-inflammatory signature in OACs (Fig. 3G, Table 1). To begin the exploration of this pro-inflammatory signature, we performed a KEGG enrichment analysis. Among the KEGG-enriched pathways identified using this list of 56 DEPs/DEGs, some were expected, such as the “NF-κB signaling pathway” [10] and the “HIF-1 signaling pathway” [29], whereas others, such as “Antigen presentation” [30] and “Ferroptosis” [31], were more recently identified in the context of OA inflammation (Fig. 3H).

**Table 1:** List of the 56 common DEG/DEPS defining the canonical OAC pro-inflammatory signature. Summarized log_2_(Fold-Change) and associated *p*-value for the 56 targets that define the OAC pro-inflammatory signature are shown. The first 21 genes, in bold, are also differentially expressed in NCs in response to either IL-1b or TNF

Name	OAC: Transcriptomics				OAC: Proteomics				NC: Transcriptomics			
	TNF		IL-1b		TNF		IL-1b		TNF		IL-1b	
	**Log2(FC)**	**Adj. *p*-value**	**Log2(FC)**	**Adj. *p*-value**	**Log2(FC)**	***P*-value**	**Log2(FC)**	***P*-value**	**Log2(FC)**	**Adj. *p*-value**	**Log2(FC)**	**Adj. *p*-value**
***AKR1B1***	**3.89**	**7.46E-64**	**2.63**	**7.13E-27**	**0.78**	**8.80E-04**	**0.64**	**1.95E-03**	**2.74**	**8.23E-20**	**1.79**	**3.46E-07**
***APOL2***	**1.81**	**8.64E-13**	**1.63**	**8.64E-10**	**0.87**	**1.75E-02**	**0.74**	**4.98E-02**	**1.25**	**5.75E-05**	**0.77**	**9.05E-02**
***B2M***	**1.37**	**1.58E-09**	**1.69**	**2.72E-13**	**0.68**	**1.38E-02**	**0.49**	**4.98E-02**	**1.00**	**6.66E-07**	**0.77**	**1.47E-03**
***CD82***	**3.41**	**1.56E-32**	**3.62**	**3.42E-34**	**1.17**	**1.54E-02**	**2.00**	**5.21E-03**	**2.10**	**3.35E-04**	**1.88**	**6.01E-03**
***DNAJA1***	**0.92**	**6.91E-07**	**1.07**	**1.02E-08**	**0.44**	**1.75E-02**	**0.44**	**1.75E-02**	**0.86**	**2.96E-04**	**0.73**	**9.26E-03**
***EGFR***	**1.59**	**3.85E-11**	**1.42**	**2.63E-08**	**1.26**	**5.99E-03**	**1.26**	**5.99E-03**	**1.69**	**3.96E-03**	**1.31**	**9.11E-02**
***FNDC3B***	**0.92**	**4.46E-06**	**1.42**	**2.39E-13**	**0.41**	**1.48E-02**	**0.45**	**8.85E-03**	**0.79**	**4.74E-02**	**1.39**	**3.04E-05**
***FTH1***	**2.44**	**4.35E-36**	**2.72**	**2.20E-41**	**0.63**	**1.38E-02**	**0.58**	**3.31E-03**	**1.70**	**5.55E-14**	**1.71**	**3.26E-12**
***GBP2***	**2.30**	**2.48E-08**	**3.81**	**4.48E-21**	**2.24**	**6.37E-04**	**2.65**	**2.75E-04**	**2.01**	**4.37E-04**	**1.56**	**3.23E-02**
***GFPT2***	**1.08**	**1.16E-07**	**2.90**	**1.81E-58**	**0.52**	**2.98E-03**	**0.75**	**6.04E-05**	**1.16**	**1.72E-02**	**2.17**	**1.85E-07**
***HLA-B***	**2.21**	**1.93E-12**	**2.10**	**1.87E-10**	**0.88**	**2.97E-03**	**0.69**	**3.81E-02**	**1.67**	**2.58E-08**	**1.08**	**5.39E-03**
***ICAM1***	**4.65**	**1.56E-23**	**4.62**	**2.12E-21**	**4.82**	**1.21E-03**	**4.79**	**2.31E-03**	**2.52**	**7.06E-07**	**1.45**	**4.32E-02**
***ISG20***	**3.71**	**1.67E-19**	**4.08**	**7.24E-22**	**1.46**	**5.99E-03**	**1.46**	**5.99E-03**	**2.68**	**9.94E-09**	**1.38**	**4.71E-02**
***PSME1***	**2.19**	**4.14E-25**	**1.74**	**9.07E-15**	**0.68**	**2.02E-03**	**0.72**	**2.24E-03**	**1.68**	**2.98E-12**	**0.87**	**7.49E-03**
***PSME2***	**2.35**	**4.83E-28**	**1.81**	**1.95E-15**	**0.36**	**1.34E-02**	**0.36**	**1.63E-02**	**1.82**	**1.82E-17**	**0.81**	**6.13E-03**
***PTGES***	**3.41**	**2.87E-33**	**4.55**	**2.64E-56**	**1.12**	**4.49E-02**	**1.32**	**1.30E-02**	**3.09**	**1.23E-10**	**3.79**	**4.04E-14**
***SERPINE2***	**2.21**	**1.16E-32**	**2.92**	**2.00E-52**	**0.69**	**1.49E-02**	**1.04**	**2.09E-04**	**1.41**	**8.09E-03**	**1.94**	**1.22E-04**
***SLC39A14***	**2.44**	**6.94E-28**	**2.98**	**3.84E-38**	**0.87**	**1.89E-03**	**0.82**	**2.23E-03**	**1.99**	**2.65E-09**	**2.35**	**2.72E-11**
***SOD2***	**4.18**	**2.90E-42**	**4.65**	**4.83E-48**	**1.19**	**5.62E-03**	**1.32**	**2.56E-03**	**3.42**	**6.68E-18**	**3.46**	**1.02E-15**
***STAT5A***	**0.84**	**1.25E-02**	**1.37**	**1.44E-06**	**1.81**	**3.24E-03**	**1.91**	**1.61E-03**	**1.56**	**8.47E-05**	**1.02**	**9.38E-02**
***TNFAIP2***	**4.08**	**5.05E-25**	**4.15**	**5.59E-24**	**3.29**	**3.57E-04**	**3.36**	**4.34E-05**	**3.29**	**1.32E-13**	**1.87**	**1.11E-03**
*ACSL3*	1.36	7.07E-06	1.68	2.01E-08	0.45	6.79E-03	0.66	1.08E-03				
*ALCAM*	2.59	2.28E-14	1.92	1.64E-07	1.26	5.99E-03	1.14	5.42E-03				
*ANKH*	-1.08	1.83E-03	-1.00	4.78E-03	-0.68	1.38E-02	-1.19	2.90E-03				
*ATP2B1*	2.20	1.60E-12	3.00	1.86E-21	0.41	2.36E-02	0.38	5.21E-03				
*CRTAC1*	1.50	3.92E-05	2.43	1.99E-12	1.32	1.14E-03	1.87	6.51E-05			2.09	1.51E-02
*DTX3L*	2.17	8.01E-19	1.80	4.20E-12	1.26	5.99E-03	1.00	1.75E-02	1.78	6.66E-07		
*FAM162A*	1.93	4.31E-09	2.22	3.94E-11	0.87	1.75E-02	0.87	1.75E-02				
*GBP1*	2.69	3.78E-14	4.10	1.24E-30	1.65	1.24E-04	1.72	2.85E-05	1.55	4.72E-04		
*HK2*	1.91	1.40E-08	2.92	2.26E-19	0.66	1.37E-03	0.66	1.37E-03				
*HLA-A*	1.46	2.42E-04	1.41	4.76E-04	0.81	4.42E-03	0.48	4.39E-02	1.39	4.62E-02		
*HLA-C*	1.60	3.96E-10	1.53	1.02E-08	0.94	1.98E-03	0.66	5.25E-03	1.36	8.01E-04		
*IFI35*	2.78	1.12E-25	1.98	3.46E-12	2.00	6.43E-04	1.38	1.67E-02	2.27	5.49E-08		
*IFIT3*	4.78	7.25E-53	3.41	4.73E-25	3.05	1.61E-04	2.10	2.92E-02	3.63	1.72E-12		
*MMP3*	2.95	3.06E-20	4.05	9.13E-35	4.00	1.25E-04	4.49	2.77E-05			2.35	2.48E-02
*NFIA*	-1.61	8.43E-08	-2.01	2.06E-10	-0.65	3.00E-02	-1.14	5.42E-03				
*NFKB1*	1.54	5.20E-10	2.44	7.06E-25	1.00	1.50E-02	1.13	1.08E-02	1.42	7.66E-04		
*NFKB2*	2.71	1.28E-03	2.93	3.25E-04	1.91	1.61E-03	1.91	1.61E-03	2.03	8.39E-02		
*NMI*	1.81	1.36E-14	1.97	2.02E-16	1.00	1.38E-02	0.74	4.98E-02	1.47	8.92E-05		
*NOS2*	4.88	4.95E-20	5.43	7.54E-23	5.95	4.49E-03	6.32	2.45E-04	2.26	6.64E-02		
*PLSCR1*	2.30	3.91E-20	1.55	1.28E-08	1.00	1.38E-02	1.00	1.38E-02	1.92	4.17E-08		
*PML*	2.02	1.20E-06	1.86	1.30E-05	1.10	4.29E-03	1.15	3.60E-05	1.17	8.78E-02		
*PSMB9*	3.21	4.05E-29	3.06	2.19E-25	0.83	5.99E-03	0.74	5.42E-03	2.55	1.61E-07		
*PTGS2*	1.71	7.32E-05	5.85	1.80E-51	1.46	5.99E-03	4.83	6.86E-05			2.51	1.37E-03
*SLC16A3*	1.03	8.98E-02	1.08	5.33E-02	0.55	4.36E-02	0.62	3.28E-02				
*SLC2A1*	3.39	1.03E-16	3.79	2.26E-19	2.29	2.83E-03	2.39	8.60E-05				
*SLC3A2*	0.72	2.46E-03	1.21	1.23E-08	0.34	1.23E-02	0.56	4.47E-04				
*SMS*	1.26	3.16E-08	2.65	1.92E-35	0.29	4.98E-02	0.35	1.75E-02				
*TAP1*	3.09	5.86E-16	3.42	7.08E-19	2.17	1.08E-05	1.74	1.21E-03	2.11	3.63E-04		
*TAP2*	2.47	1.19E-12	2.71	9.54E-15	1.91	1.61E-03	1.32	1.38E-02	1.25	1.13E-02		
*TENT5A*	1.31	7.75E-11	1.47	1.16E-12	1.32	1.38E-02	1.17	1.54E-02				
*THBS1*	-0.68	7.20E-04	-0.87	8.91E-06	-0.74	1.08E-02	-1.47	3.54E-03				
*TRIM25*	2.55	3.08E-10	1.82	3.67E-05	1.19	1.82E-02	0.89	4.36E-02	1.16	1.50E-02		
*UACA*	-1.39	2.47E-11	-1.20	5.22E-08	-1.49	3.28E-02	-1.81	3.05E-02			-1.17	1.89E-02
*VCAM1*	3.63	8.57E-44	1.97	4.06E-12	3.64	9.87E-06	2.66	3.08E-03	2.25	3.14E-07		
*WARS1*	1.98	4.16E-11	2.93	3.19E-22	0.41	4.17E-02	0.39	3.73E-02	0.86	4.46E-02		

### OACs exhibit disease-specific metabolic features following pro-inflammatory exposure

**Fig. 4:**
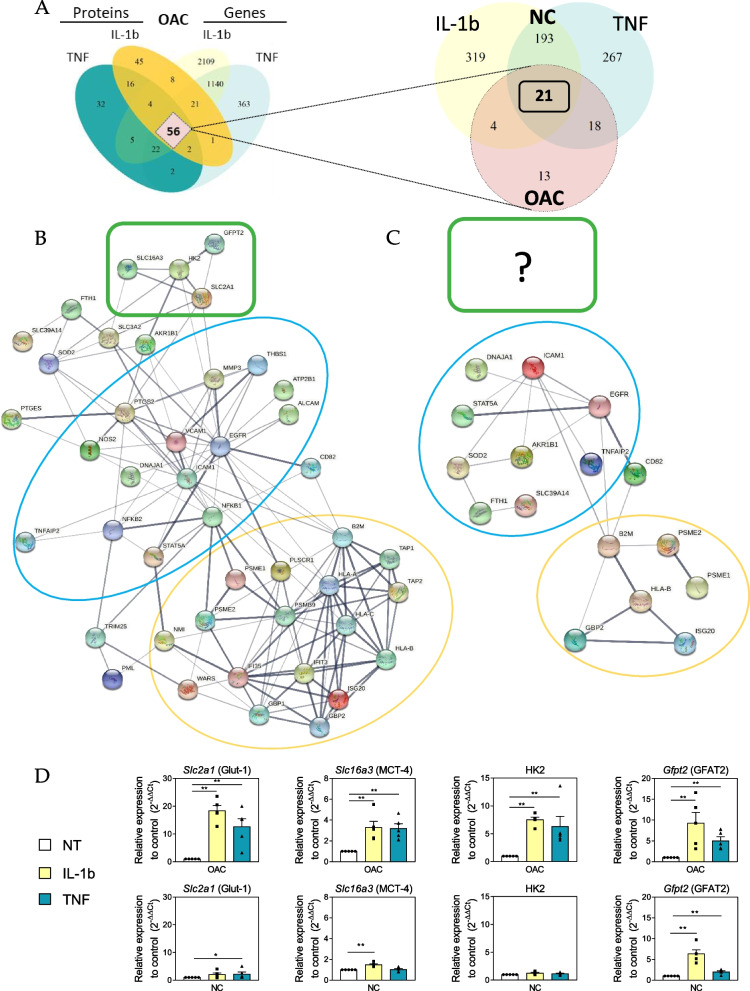
Identification of a specific feature of OACs. (**a**) Venn diagram showing the 21 common DEGs among the 56 defining the pro-inflammatory signature of OACs and TNF and IL-1b responses in NCs. (**b**) Protein–protein network analysis using the pro-inflammatory signature identified in OACs (**c**) and using the 21 DEGs also found in response to IL-1b and TNF treatments in NCs. (**b**, **c**) Three interacting groups were revealed by this analysis: The blue circle group was characterized by common pro-inflammatory targets, the yellow circle group was associated with proteins involved in antigen presentation, and the green box group was composed of metabolism-related proteins. (**d**) RT-qPCR analysis of Glut-1, MCT-4, HK2, and GFAT2 expression in OACs and NCs after 24 h of IL-1b or TNF treatment; *P < 0.05, ** *P* < 0.01 using the Mann–Whitney test; *N* = 5 per group. Values are expressed as means ± SEM

Following the description of the canonical pro-inflammatory signature characterizing OACs, we wished to determine whether the same pro-inflammatory signature could also be observed in non-OA chondrocytes. To address this question, the 56 matched DEPs/DEGs defining the canonical pro-inflammatory signature of OACs were searched in the 214 common DEGs identified in NCs in response to IL-1b or TNF. These 214 DEGs comprised 21 of the 56 DEGs/DEPs identified in OACs (Table 1, bold genes) (Fig. 4A). To further characterize the differences in the inflammatory responses of OACs versus NCs, protein–protein networks were constructed, using either the 56 DEGs/DEPs of OACs (Fig. 4B) or the 21 DEGs retrieved in NCs (Fig. 4C). Interestingly, the 56 DEGs/DEPs characterizing the pro-inflammatory signature of OACs revealed three interacting groups of proteins (Fig. 4B): one group was characterized by common pro-inflammatory targets (Blue circle: SOD2, ICAM-1, EGFR, etc.), another was associated with proteins involved in antigen presentation (Yellow circle: HLA-B, B2M, PSME2, etc.), and a third group was composed of metabolism-related proteins (Green box: SLC2A1, HK2, SLC16A3, and GFPT2). A similar analysis using the 21 DEGs characterizing the NCs response to IL-1b and TNF (Fig. 4C) demonstrated that, while the common pro-inflammatory targets (Blue circle) and antigen presentation (Yellow circle) groups were shared between both the NC and OAC pro-inflammatory responses, the group related to metabolism was specific to the OAC response. This metabolism group is composed of four proteins: Glut-1 (Solute Carrier Family 2 Member 1 – *Slc2a1*), a glucose transporter; MCT-4 (Solute Carrier Family 16 Member 3 – *Slc16a3*), a lactate/H^+^ exporter; HK2 (hexokinase 2), the enzyme catalyzing the first step in glycolysis; and GFAT2 (Glutamine-Fructose-6-Phosphate Transaminase 2 – *Gfpt2*), the rate-limiting enzyme of the hexosamine biosynthesis pathway (HBP). Among these, *Gfpt2* expression was also increased in NCs, but the expression of Glut-1, MCT-4, and HK2 was specifically modulated in OACs (Table 1). Using RT-qPCR, we then validated the overexpression of Glut-1, MCT-4, and HK2 in OACs by both cytokine treatments as well as the overexpression of GFAT2 in response to either IL-1b or TNF in OACs and NCs (Fig. 4D).

**Fig. 5:**
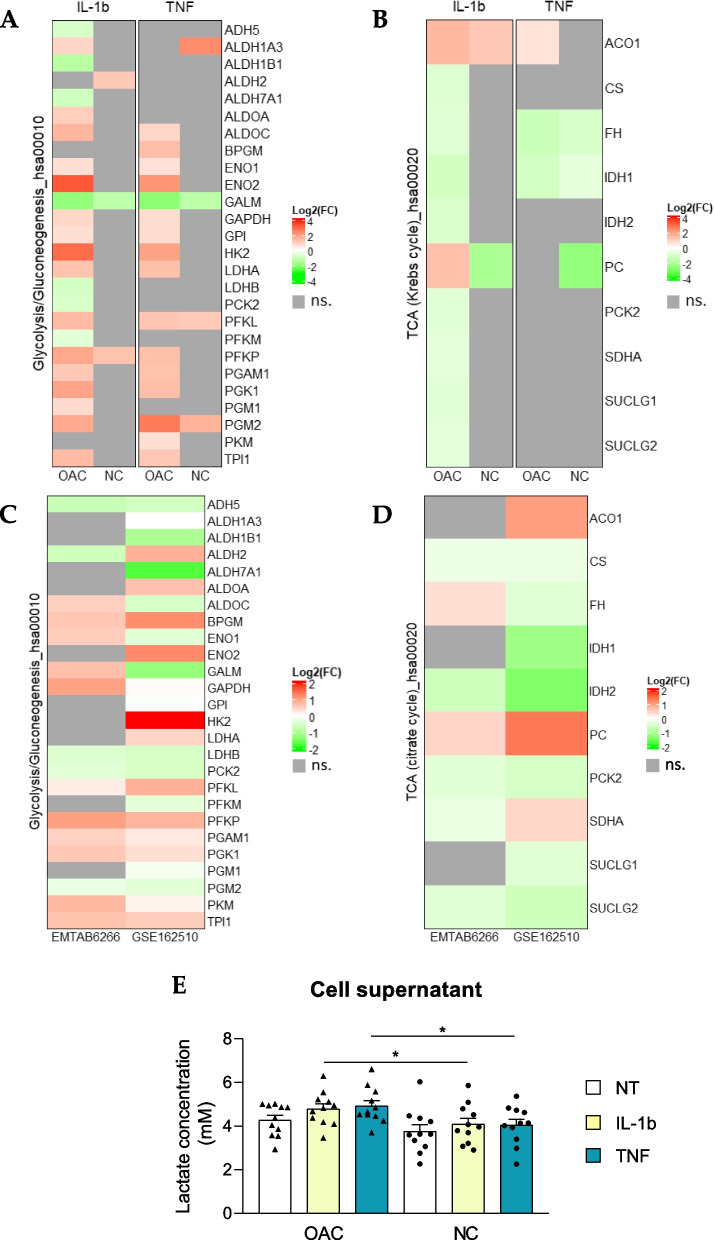
Evidence of an OAC-specific metabolic switch. (**a**, **b**) Heatmaps displaying the log_2_(Fold-Change) in OACs or NCs for genes annotated in the KEGG database for (**a**) glycolysis/neo-glucogenesis and (**b**) the Krebs cycle (TCA). Heatmaps from the public transcriptome dataset (E-MTAB-6266 and GSE162510) highlighting glycolysis (**c**) and TCA (**d**) gene modulation. **e** Lactate measurement in OAC (*N* = 11) and NC (*N* = 11) supernatants; ns.: not significant; *P < 0.05 using the Mann–Whitney test; *N* = 11 per group. Values are expressed as means ± SEM

Since Glut-1, MCT-4, and HK2 are involved in the key steps controlling glycolytic flux [32], we used the glycolysis/gluconeogenesis-related KEGG pathway annotation (*hsa00010)* to construct heatmaps and visualize dysregulations occurring in OACs versus NCs in response to pro-inflammatory cytokines (Fig. 5A). This analysis revealed an overall increase in the expression of glycolysis enzymes specifically in OACs treated with either IL-1b or TNF (Fig. 5A). As the final product of glycolysis is pyruvate, which is a metabolite that can initiate the tricarboxylic acid cycle (TCA, or citrate/Krebs cycle), we examined TCA-related genes using unsupervised heatmap analysis based on the TCA-related KEGG annotation (*hsa00020*). The TCA heatmap showed an overall decrease in TCA-related genes in OACs but not in NCs (Fig. 5B). To strengthen our results, glycolytic and TCA gene dysregulation were also explored in two public datasets: GSE162510, which explored the transcriptomic changes upon IL-1b treatment, and E-MTAB-6266, which compared gene expression between the cartilage of OA knees and the cartilage of healthy knees from multi-organ donors as a control. Interestingly, both datasets confirmed the expression profile highlighted in our study, with overexpression of the genes associated with glycolysis (Fig. 5C), and downregulation of the genes related to TCA (Fig. 5D) in OA conditions compared to the controls (untreated chondrocytes or chondrocytes from non-OA joints).

To better understand the impact of this glycolytic increase on the overall cellular metabolism, we also explored other metabolic pathways directly related to glycolysis, such as the HBP and the pentose phosphate pathway. No regulation as clear and specific as the one observed for glycolysis was revealed for these pathways (Figure S2A, B). Moreover, in keeping with the increase observed in the expression of glycolysis enzymes and the decrease in the expression of TCA enzymes, our results revealed overexpression of *LDHA* and, as previously described, overexpression of MCT-4 (*Slc16a3)* at both the transcriptomic and proteomic levels in response to either IL-1b or TNF in OACs (Fig. 5A, Table 1). Based on these observations, we examined whether lactate was abnormally secreted in OACs treated with IL-1b or TNF, and we found a significant increase in the extracellular lactate concentrations in treated OACs compared to NCs (Fig. 5E). These data suggest a metabolic shift of OACs in response to pro-inflammatory cytokines.

### Real-time cell metabolic analysis confirmed induction of OAC-specific metabolism by pro-inflammatory stimuli

**Fig. 6:**
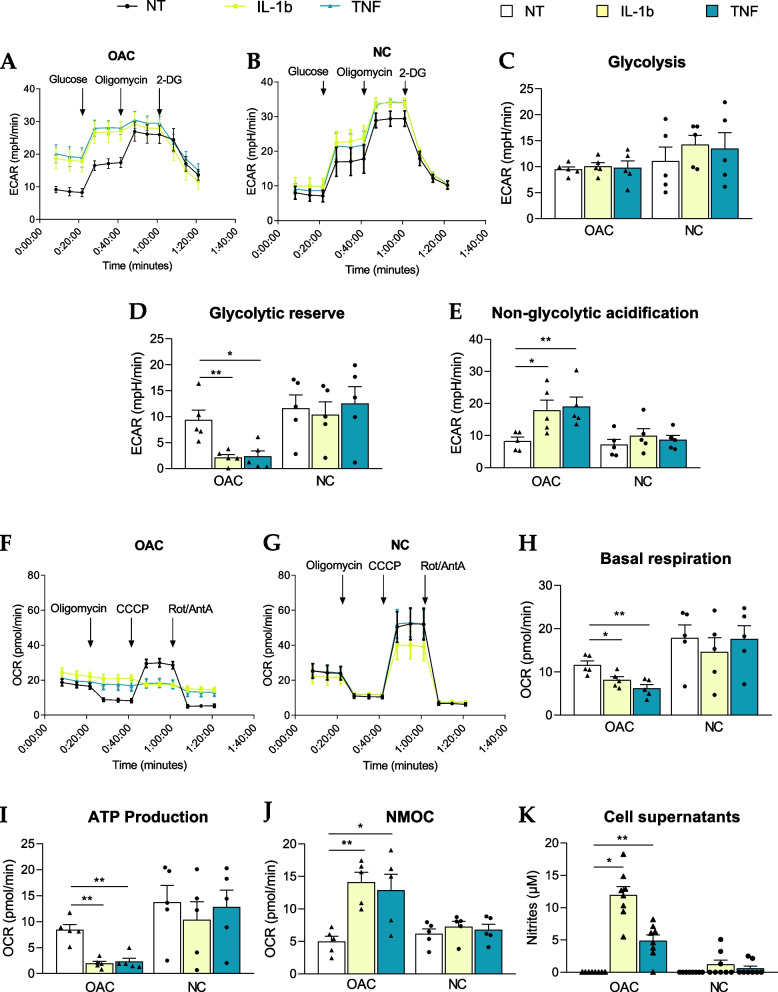
Investigation of OAC-specific functional metabolic changes. **a**-**e** Seahorse® assay evaluating ECAR measurements in the glycolysis stress test in OACs (**a**) and NCs (**b**). **c** Quantification of glycolysis, (**d**) glycolytic reserve, and (**e**) non-glycolytic acidification from the glycolysis stress tests. **f**-**j**) Seahorse® assay evaluating OCR measurements in the mitochondrial stress assay in OACs (**f**) and NCs (**g**). (**h**) Quantification of basal respiration, (**i**) ATP production, and (**j**) non-mitochondrial oxygen consumption (NMOC) from the mitochondrial stress assay. **k** Nitrite measurements in OAC and NC supernatants; *P < 0.05, **P < 0.01 or ***P < 0.001 using the Mann–Whitney test; *N* = 5 (Seahorse® assays) or *N* = 8 (nitrite measurements) per group. Values are expressed as means ± SEM

To strengthen our multi-omic analysis, we then examined whether the metabolic shift has a functional impact on cellular metabolism. This was addressed by performing Seahorse® real-time metabolic assays. We evaluated both the extracellular acidification rate (ECAR) during a glycolytic stress test and the oxygen consumption rate (OCR) during a mitochondrial stress test in both OACs and NCs upon IL-1b and TNF treatments. As expected, according to previous results presented in Fig. 5, OACs and NCs had distinct metabolic profiles. Indeed, the ECAR measured during the glycolytic stress assay revealed an altered profile of OACs treated with IL-1b or TNF compared with untreated cells, while this was not observed in NCs (Fig. 6A, B). Unexpectedly, OAC and NC samples did not exhibit differences in glycolysis levels, irrespective of the applied treatments (Fig. 6C). However, pro-inflammatory stimuli significantly decreased the glycolytic reserve of OACs but not NCs (Fig. 6D). Interestingly, non-glycolytic acidification was increased only in treated OAC samples but not in treated NC samples (Fig. 6E), suggesting that other pathways are involved in the acidification of the OAC environment.

In parallel, the mitochondrial stress assay revealed altered oxidative phosphorylation (OXPHOS) in response to IL-1b or TNF in OACs (Fig. 6F) compared to the expected NC profiles (Fig. 6G). Consequently, the OAC basal respiration level was significantly decreased by pro-inflammatory stimuli (Fig. 6H), and it was associated with a significant reduction in ATP production by OXPHOS (F ig. 6I). Consistent with our RNA-seq data, pro-inflammatory stimuli did not alter OXPHOS or ATP production in NCs (Fig. 6H-J). Interestingly, non-mitochondrial O_2_ consumption (NMOC) increased specifically in OACs upon IL-1b and TNF treatments (Fig. 6J). An increased NMOC suggests that OACs treated with cytokines consumed O_2_ in other ways than OXPHOS, such as NO and/or ROS production. Of note, the nitrite (NO_2_^−^) concentration was specifically increased in OAC supernatants by pro-inflammatory stimuli (Fig. 6K). Since nitrite production resulted from NO oxidation, part of the increased NMOC observed in OACs but not in NCs could, therefore, be explained by NO production.

## Materials and methods

### Experimental design

Primary human chondrocytes, isolated from osteoarthritic knees of advanced OA patients (OACs) and vertebral transverse costal facet joints of non-OA patients (NCs). For histological validation, cartilage samples were paraffin-embedded and stained with Safranin-O. Isolated OA chondrocytes (OACs) and non-OA chondrocytes (NCs) were treated for 24 h with or without 1 ng/mL of IL-1b or 25 ng/mL of TNF or 50 ng/mL of IL-6/IL-6 soluble receptor (IL-6). Sequencing by 3’ SRP was performed on OAC and NC samples. Moreover, mass-spectrometry was performed on OACs. The pro-inflammatory signature was defined with matched transcripts and proteins dysregulated by IL-1b or TNF treatments. NC transcriptomics data were then compared to the OAC pro-inflammatory signature (Fig. 1).

### Cell culture

#### Human chondrocytes

Primary human chondrocytes were isolated from osteoarthritic joints of advanced OA patients undergoing total knee surgery (OACs, 71–81 years old, patient number indicated in the figure legends) and from vertebral transverse costal facet joints of non-OA patients undergoing scoliosis surgery (NCs, patient number indicated in the figure legends*,* 15–21 years old). According to the Declaration of Helsinki, all human articular cartilage samples were harvested from patients after they had provided informed consent*.* This study was carried out following the recommendation of the Committee for Person Protection of Pays de La Loire and approved by the French Ministry of Higher Education and Research (registration number: DC-2017–2987). Briefly, cartilage was cut into small slices and placed in Hanks' Balanced Salt Solution (HBSS, L0606-500, Biowest) supplemented with 10% penicillin–streptomycin solution (PS, 1000 U/mL, 15,140,122, Thermo Fisher Scientific) at 37 °C in a humidified atmosphere with 5% CO_2_ for up to 5 h. Then, small slices were digested for 30 min in 0.2% collagenase (125 U/mg, C6885, Sigma-Aldrich), and chondrocytes were released by overnight digestion (~ 14 h) in 0.03% collagenase in high-glucose Dulbecco's Modified Eagle’s Medium (DMEM, 31,966–021, Gibco) containing 10% fetal calf serum (FCS, Dominique Dutscher) and 1% PS (complete medium) and passed through a 70 µm filter to remove undigested cartilage fragments. Isolated chondrocytes were seeded at a density of 6.0 × 10^4^ cells/cm^2^ for OACs and 1.5 × 10^4^ cells/cm^2^ for NCs and used at passage 1 after reaching 90% confluence.

#### Chondrocyte treatments

Primary human chondrocytes (OACs and NCs) were seeded at 6 × 10^4^ cells/cm^2^ in complete medium and cultured at 37 °C in a humidified atmosphere with 5% CO_2_. After 24 h, cytokines were added as follows: 1 ng/mL IL-1b (Merck, IL038) or 25 ng/mL TNF (Miltenyi Biotec 130–094-023) or 50 ng/mL IL-6/IL-6 soluble receptor (R&D, 8954-SR-025) for 24 h. At the end of the treatments, the supernatants were collected and the cells were lysed before being stored immediately at -80 °C until further analysis.

For the transcriptomic analysis, total RNA was extracted from lysed chondrocytes using NucleoSpin RNA XS (740,902.50, Macherey–Nagel) according to the supplier’s instructions. The RNA yield and RNA quality were assessed for the 3’ SRP analysis using a NanoDrop™ spectrophotometer and an Agilent Bioanalyzer system (Agilent Technologies), respectively. The RNA Integrity Number was greater than 9 for each RNA sample.

For the mass spectrometry analysis, cells were trypsinized, washed with PBS, and then pelleted before being snap-frozen and stored at -80 °C until further analysis.

### Chondrocytes analysis

#### qRT-PCR

RNAs were reverse transcribed to cDNA using a Verso™ cDNA Synthesis Kit (Thermo Fisher Scientific, AB-1453/B). The diluted cDNA was then amplified with specific primers (Table 2) using the SYBR® Select Master Mix (Thermo Fisher Scientific, 4,472,897). qPCR was then performed using a CFX96™ Touch Deep Well Real-Time PCR Detection System (Bio-Rad).

**Table 2:** Specific primers used for qPCR analysis

Target gene	Forward	Reverse
Slc16a3 (MCT-4)	CCACAAGTTCTCCAGTGCCATTG	CGCCAGGATGAACACGTACATG
HK-2	GAGTTTGACCTGGATGTGGTTGC	CCTCCATGTAGCAGGCATTGCT
Slc2a1 (Glut-1)	TTGCAGGCTTCTCCAACTGGAC	CAGAACCAGGAGCACAGTGAAG
Gfpt2 (GFAT-2)	GCTCATCGTGATTGGCTGTGGA	CAACCATCACAGGAAGCTCAGTC
18S	AGCAAACCCCAACTCAACC	GTCCCTCAGAAGGGGTGAC
GusB	CGCCCTGCCTATCTGTATTC	TCCCCACAGGGAGTGTGTAG

#### Supernatants analysis

Nitrite concentrations were determined using the Griess reaction. Aliquots of 100 µL of cell supernatants were mixed with 100 µL of Griess solution (v/v mixture of a 0.1% aqueous solution of naphthylenediamine dihydrochloride and a 1% solution of sulfanilamide in 5% H_3_PO_4_). The absorbance was then measured at 550 nm, and the nitrite concentration was determined using a standard range of nitrites diluted in complete medium. Lactate concentrations were determined using a Lactate Assay Kit (Sigma-Aldrich, MAK064) with 1 µL of the cell supernatants following the manufacturer’s instructions.

### Measurement of cellular metabolism by Seahorse® assay

Primary human chondrocytes (OACs or NCs) were seeded in Seahorse® XFp plates at 15 000 cells per well (1.3 × 10^5^ cells/cm^2^) and treated with IL-1b (1 ng/mL) or TNF (25 ng/mL). After 24 h, the complete medium was replaced with DMEM (Sigma-Aldrich, 5030) with 1% P/S and glutamine (4 mM) for the glycolytic stress assay or glutamine (4 mM), glucose (25 mM), and pyruvate (1 mM) for the mitochondrial stress test. The plate was then preincubated at 37 °C for 45 min in the absence of CO_2_. The glycolytic stress assay consisted of the sequential addition of glucose (25 mM), oligomycin (1 µM), and 2-deoxy-D-glucose (2-DG, 50 mM) according to the manufacturer's instructions. The mitochondrial stress test consisted of the sequential addition of oligomycin (1 µM), carbonylcyanide-3-chlorophenylhydrazone (CCCP, 1 µM), and rotenone/antimycin A (1 µM each). Measurements of the extracellular acidification rate (ECAR) and the oxygen consumption rate (OCR) were performed every 5 min before and after the addition of the various drugs as indicated in the figures. The data analysis was performed using Wave software (Agilent).

### 3’ Seq-RNA Profiling (3’ SRP)

#### Preparation of the sequencing library

The 3'seq-RNA profiling was performed as described by Charpentier et al., 2021 and (48). Samples were randomly plated and diluted to a concentration of 2.5 ng/μL to construct the library. The library was prepared from 10 ng of total RNA in 4 µL. The mRNA poly-A tails were tagged with universal adapters, well-specific barcodes, and unique molecular identifiers (UMIs) during template-switching reverse transcriptase (Thermo Scientific™, EP0751, Maxima H Minus Reverse Transcriptase). Barcoded cDNAs from 96 samples were then pooled and purified using a Zymo kit. cDNAs were treated with exonuclease I (NEB, M0293S) before being amplified (12 cycles) and fragmented using a transposon-fragmentation approach that enriched for 3′ ends of cDNA. The fragments were size controlled on a 2200 TapeStation system (Agilent Technologies) to form a library of 350–800 bp in length. Finally, the library was validated with fragments of approximately 484 pb length and with 38.5 nM of properly tagged cDNA.

#### Sequencing and primary analysis

Sequencing and primary analysis were performed according to Charpentier et al., 2021. Briefly, the library was sequenced on a NovaSeq 6000 using NovaSeq 6000 SP Reagent Kit 100 cycles (#20,027,464, Illumina) with 17*-8–105* cycle reads (* addition of one cycle according to Illumina’s recommendation). Bioinformatics steps were performed using a Snakemake (49) pipeline. Sample sequences were demultiplexed using a python script, and raw paired-end fastq files were transformed into a single-end fastq file for each sample and aligned on the Ensembl transcriptome hg38 using bwa.

#### Public datasets analysis

Two public datasets, E-MTAB-6266 and GSE162510, were uploaded to explore the regulation of metabolism-associated genes. E-MTAB-6266 compared gene expression between the cartilage of 58 OA knees and 10 non-OA knees. GSE162510 explored the transcriptomic changes occurring in chondrocytes from 10 OA patients following 24 h of IL-1b treatment (0.2 ng/mL). A differential expression analysis table of the E-MTAB-6266 public dataset was uploaded from Soul et al. [33], while GSE162510 was uploaded with the R package GEO query (2.62.2). Data visualization was then performed as previously described.

### Mass-spectrometry

#### Proteome sample preparation and tandem mass spectrometry

Cell pellets were dissolved in 25 µL of LDS buffer (26.5 mM Tris HCl, 35.25 mM Tris base, 0.5% LDS, 2.5% glycerol, 0.13 mM EDTA, supplemented with 5% beta-mercaptoethanol) per mg of pellet. Samples were heated for 5 min at 99 °C in a thermomixer (Eppendorf). For each sample, a volume of 20 µL (i.e., 10 µg of protein) was subjected to a 5 min denaturing electrophoresis on a NuPAGE 4%–12% gradient gel with MES SDS as the running buffer (50 mM MES ([2-(N-morpholino) ethane sulfonic acid), 50 mM Tris Base, 0.1% SDS, 1 mM EDTA, pH 7.3). Following electrophoresis, the gel was briefly washed with Milli-Q® water, stained with SimplyBlue™ SafeStain (Thermo Fisher Scientific) for 5 min to visualize proteins, and then washed extensively with Milli-Q® water. Each proteome was extracted as a single polyacrylamide band with a volume of approximately 100 µL. Each sample was processed as previously described (50) and then proteolyzed with Trypsin Gold (Promega) in 50 mM NH_4_HCO_3_ in the presence of ProteaseMAX™ detergent (Promega). A volume of 10 µL of the resulting peptide mixture (45 µL) was injected into a nanoscale C18 PepMap™ 100 capillary column (3 µm, 100 Å, 75 µm internal diameter × 50 cm length, LC Packings) mounted with a desalting pre-column and resolved with a 65-min gradient of acetonitrile (4.8%–24.8% in 60 min followed by 24.8%–40% in 5 min), 0.1% formic acid, at a flow rate of 0.25 µL/min. The peptides resolved by reverse-phase chromatography were analyzed by tandem mass spectrometry with an Exploris™ 480 mass spectrometer (Thermo Fisher Scientific) connected directly to the column exit. The instrument was operated in data-dependent acquisition mode, with a full scan of peptide ions acquired at a resolution of 120,000 from m/z 350 to 1500 and with a dynamic exclusion of 10 s. Each MS scan was followed by high-energy collisional dissociation and MS/MS scans at a resolution of 15,000 on the 20 most abundant precursor ions identified within the full scan, selecting only ions with a charge of 2^+^ or 3^+^.

#### Label-free shotgun proteomic interpretation

MS/MS spectra were assigned to peptide sequences by the MASCOT Daemon 2.3.2 search engine (Matrix Science) in follow-up mode using first the contaminant database described by Pereira et al. (51) including the 23 most abundant proteins from fetal calf serum, and then the SwissProt *Homo sapiens* database (20,396 polypeptide sequences). Standard search parameters included: trypsin as a proteolytic enzyme with two possible miss-cleavages at maximum, tolerances of 5 ppm and 0.02 Da for the MS and MS/MS signals, respectively, oxidation of methionine, deamidation of glutamine and asparagine, and acetylation of N-termini, as variable modifications, carbamidomethylation of cysteine as fixed modification, and a peptide p-value below 0.01. A protein was considered validated when at least two different peptides were detected, resulting in a protein identification false discovery rate below 1% as verified with a reverse decoy database search. To account for possible genome heterogeneity between patients, only peptides common between all patients (at least identified once in a patient sample) were used for the comparative proteomics. Spectral counts corresponding to the number of MS/MS spectra assigned per protein were used as a proxy for the abundance of the proteins in each condition, considering only unambiguous peptides (listed only once in the database).

### Omic data processing

#### Differential expression analyses

Aligned reads were parsed, and UMIs were counted for each gene in each sample to create an expression matrix containing the absolute abundance of mRNAs in all samples. Reads aligned on multiple genes or containing more than three mismatches with the reference were discarded. The expression matrix was normalized and differentially expressed genes (DEG) were searched using the R package DeSeq2 (52) (thresholds used: adjusted *p*-value < 0.01 and log_2_(Fold-Change) > 0.58). Supplementary R packages compiled with R version 4.1.1 were used to generate data visualization: ggplot2 (3.3.5), VennDiagram (1.7.3), enrichR (3.0), and ComplexHeatmap (2.8.0).

#### Protein–protein network

Protein–protein network analysis, using the 56 DEGs/DEPs identified in OACs and the 21 DEGs of NCs, were determined using the Search Tool for the Retrieval of Interacting Genes (STRING) database version 11.5. All available interactions were mapped with an interaction score cutoff of 0.4, and unconnected nodes were removed.

### Statistical analysis

The statistical analyses used in the transcriptomic analysis were performed using the R software package DESeq2 (52) with a Benjamini & Hochberg correction. The PCA and biplots were created using the prcomp function from the stats package within R statistics (version 4.1.1) and the factoextra R package (1.0.7). Differences in continuous variables were assessed using the non-parametric Mann–Whitney test (n < 30), and p-values less than 0.05 were considered to be statistically significant. The statistical analyses were performed using GraphPad Prism 8.0v software.

## Discussion

Using a multi-omics approach, this study presents, a broad description of IL-1b and TNF responses of chondrocytes from OA joints compared to chondrocytes from non-OA joints. Our multi-omics approach based on a 3’-seq RNA analysis and mass spectrometry analysis of OAC lysates identified 56 targets of interest that define a canonical pro-inflammatory signature of OACs induced in response to either IL-1b or TNF. The protein–protein network analysis of the 56 targets identified in the canonical pro-inflammatory signature of OACs defined three main interactome groups. By comparing the OAC canonical pro-inflammatory signature to NCs, we identified that the group corresponding to metabolism was absent in the pro-inflammatory response of NCs.

This metabolism group comprises four proteins: Glut-1, HK2, MCT-4, and GFAT2. Thus, a special focus on energetic metabolism pathways such as glycolysis and TCA at the transcriptomic level revealed that the expression of glycolytic enzymes and TCA enzymes are inversely regulated in OACs. Indeed, our study, showed, that the expression of most genes involved in glycolysis is increased whereas the expression of those related to TCA is decreased in OACs upon exposure to IL-1b or TNF. This increased expression of glycolytic genes such as Glut-1, HK2, or LDHA has been previously described in the pro-inflammatory response of OA and non-OA chondrocytes [34–37]. However, in our study, no significant regulation of metabolism-related genes upon exposure to IL-1b or TNF was observed in non-OA chondrocytes (NCs). This finding was confirmed by a public data set obtained from chondrocytes isolated from knees with OA and healthy knees from age-matched multi-organ donors (E-MTAB-6266) [33]. Interestingly, promising studies have also highlighted the potential benefit of glycolysis inhibition on the inflammatory response of chondrocytes [38, 39].

We further examined the bioenergetic profiles of chondrocytes using real-time cell metabolic assays. Interestingly, we confirmed the functional-specific impact on OACs of the metabolic gene modulations identified in our transcriptomic analysis. Indeed, Seahorse® glycolytic stress assay showed that pro-inflammatory conditions forced OACs to use glycolysis at their full capacity, which was not observed in NCs. Furthermore, a significant increase in non-glycolytic acidification was specifically observed in OACs upon treatment with IL-1b or TNF. An acidic microenvironment is a hallmark of inflamed tissue as well as cartilage from OA patients [40] and has been associated with decreased glycosaminoglycan production and increased MMP activity in cartilage [41]. Microenvironment acidification could be due to TCA activity, glycogenolysis, or lactate export (linked to glycolysis) [42]. In our study, the increased expression of LDHA and MCT-4 expression suggests enhanced lactate secretion in OACs following exposure to pro-inflammatory stimuli. We confirmed that lactate concentrations increased in the supernatants of OACs compared to NCs under pro-inflammatory conditions. An increase in synovial fluid lactate levels has already been associated with inflammatory joint diseases, such as rheumatoid arthritis [43], and in OA animal models [44, 45]. Altogether, these data confirmed that disease-specific metabolic dysregulation characterizes OACs.

In parallel, our results showing decreased expression in TCA-related genes, basal respiration, and ATP production suggest an impaired mitochondrial function in OACs upon IL-1b and TNF treatments. Consistent with our data, several metabolic dysregulations have been shown previously in OA chondrocytes, such as OXPHOS impairments [18, 19, 24, 46]. Here, our study provides the additional information that pro-inflammatory stimuli exacerbate OXPHOS dysfunction in OA chondrocytes, whereas NCs appeared to be less sensitive to pro-inflammatory stimuli and thus did not exhibit metabolic alterations associated with glycolysis or OXPHOS. Contrary to our results, the previous study by Eitner et al. showed that IL-1b and TNF treatments induced much more pronounced metabolic alterations in non-OA chondrocytes compared to OA chondrocytes [24] after 48 h of treatment. However, these differences with our results could be explained by differences in treatment conditions (duration and dose), as well as in the anatomical source of chondrocytes (hip).

Interestingly, in OACs but not in NCs, both IL-1b and TNF induced an increase in NMOC associated with an increase in nitrite production, reflecting O_2_ consumption for NO production. As NO is a known chondrocyte catabolic product found in OA [47, 48], this increased nitrite production strengthens the association of these metabolic dysfunctions with pathogenic OA processes. Our observations, therefore, suggest that the inflammatory and/or catabolic environment chondrocytes experience in OA joints for several years makes them more responsive to metabolic changes induced by pro-inflammatory stimuli. Whether sustained treatment of NCs with low-grade pro-inflammatory stimuli could sensitize them to this metabolic shift merits further analysis.

Although our study focused on glycolysis and TCA due to its specificity to OACs, other pathways, such as the hexosamine biosynthetic pathway (HBP), are regulated by pro-inflammatory stimuli. This HBP produces the monosaccharide UDP-GlcNAc involved in GAG synthesis and the post-translational modification called O-GlcNacylation [49], integrates multiple metabolic pathways such as glucose, glucosamine, amino acid, fatty acid, and nucleotide metabolisms. Thus, it could also be explored in depth. Indeed, our study highlighted that the expression of several players of the HBP are up-regulated in both OAC and NC treated with IL-1b- or TNF. Unlike what is observed for glycolysis and OXPHOS, inflammation can induce a modification of the HBP in chondrocytes regardless of their status, OA or not. This suggests either that this metabolic pathway is not specifically altered in OA or that it may be altered early in the development of OA. Few studies have explored the involvement of this HBP in OA [49, 50], mainly focusing on its interplay with glucosamine, a known but controversial component in the management of OA.

Interestingly our study also highlighted a lack of relevant transcriptomic and proteomic changes by IL-6 exposure in human chondrocytes (osteoarthritic and non-osteoarthritic) under the experimental conditions tested. This result may seem surprising as studies have described a protective effect of IL-6 inhibition in preclinical models of OA [12], suggesting that IL-6 is a key signaling cytokine in joints. However, anti-IL-6 clinical trials have been ineffective in OA, highlighting the species variability in IL-6 signaling. This is consistent with our data, where a transcriptomic response to the same IL-6 treatment was observed in murine chondrocytes (530 DEGs, data not shown) but not in human chondrocytes.

The design of our study does not completely rule out the possibility that the observed differences in response to pro-inflammatory stimuli are related to the use of chondrocytes from different joints. However, all our observations were supported by additional studies using publicly available datasets. Nevertheless, since accessing a healthy knee joint is challenging, supplementation of this study with other animal models such as dogs or horses would be substantial. These future studies should also complement the pro-inflammatory signature with data on secretome and metabolome dysregulations. Despite these limitations, the main interest of our study lies in the multi-omics approach completed by a functional approach to investigate the action of two different pro-inflammatory treatments.

Our study identified a canonical pro-inflammatory signature and its specific signaling pathways in chondrocytes from OA joints compared to chondrocytes from non-OA joints. Although IL-1b or TNF treatments are widely used to mimic the pro-inflammatory component of OA, the specificity of the metabolic alterations observed only in OACs strengthens the need for a thorough study of the link between inflammation and metabolism. Indeed, our study suggests that the pathological history of OA chondrocytes (inflammation, pre-established metabolic alterations) may promote abnormal catabolic responses to stress and thus sustain joint degeneration. Finally, based on the relative resistance of NCs to metabolic shifts induced by proinflammatory stimuli, further studies may help design relevant strategies to efficiently target upstream processes to inflammation-related metabolic change.

## Supplementary Information

**Additional file 1: Fig. S1.** Comparison of OACs and NCs pro-inflammatory responses. (a,b) Venn diagram highlighting OAC and NC matched DEGs in response to (a) 1 ng/mL of IL-1b or (b) 25 ng/mL of TNF

**Additional file 2: Fig. S2.** Glycolysis-linked metabolic pathways. (a,b) Heatmaps displaying the log2 (Fold-Change) in OACs and NCs for genes associated with (a) hexosamine biosynthesis pathway and (b) pentose phosphate pathway. ns.: not significant.

## Data Availability

The mass spectrometry and proteomics data have been deposited to the ProteomeXchange Consortium via the PRIDE (53) partner repository with the dataset identifiers PXD037145 and 10.6019/PXD037145. All sequencing 3' SRP data are publicly available via the NCBI Gene Expression Omnibus (GEO) using the accession number GSE215039.
