# Supplementary figures and images for: Osteoarthritic chondrocytes undergo a glycolysis-related metabolic switch upon exposure to IL-1b or TNF
Source: Cell Commun Signal. 2023 Jun 14;21:137. doi: 10.1186/s12964-023-01150-z (PMC10265918; doi:10.1186/s12964-023-01150-z)

**A**

**IL-1b**

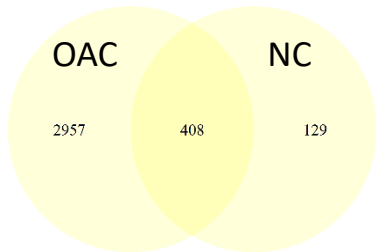

**B**

**TNF**

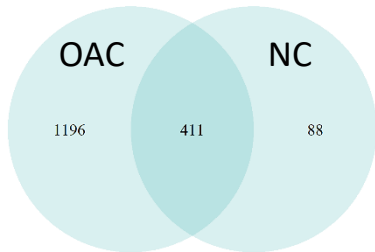

Supplement: Supplementary file 2 — Additional file 1: Fig. S1. Comparison of OACs and NCs pro-inflammatory responses. (a,b) Venn diagram highlighting OAC and NC matched DEGs in response to (a) 1 ng/mL of IL-1b or (b) 25 ng/mL of TNF [file 12964_2023_1150_MOESM1_ESM.pdf]

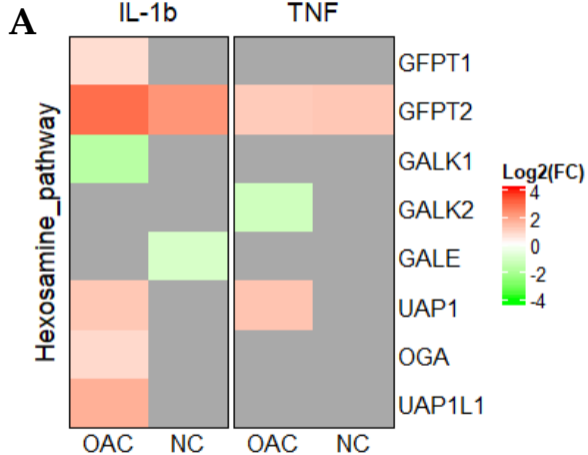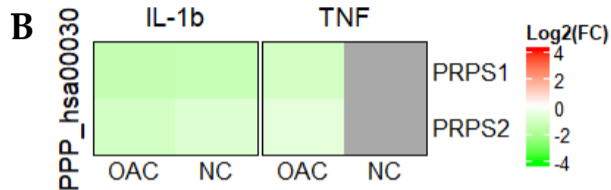

Supplement: Supplementary file 3 — Additional file 2: Fig. S2. Glycolysis-linked metabolic pathways. (a,b) Heatmaps displaying the log2 (Fold-Change) in OACs and NCs for genes associated with (a) hexosamine biosynthesis pathway and (b) pentose phosphate pathway. ns.: not significant. [file 12964_2023_1150_MOESM2_ESM.pdf]
